# Corrigendum to Trends in tracheal, bronchial and lung cancer attributed to smoking in South America: Global Burden of Disease analysis 1990-2019

**DOI:** 10.26633/RPSP.2024.65

**Published:** 2024-04-04

The *Pan American Journal of Public Health* draws the readers’ attention to an error in the following article, pointed out by the authors.

In page 2 the following section should read as follows:

## GBD database analysis

Our study included the 12 sovereign countries of SA (Argentina, Bolivia, Brazil, Chile, Colombia, Ecuador, Guyana, Paraguay, Peru, Suriname, Uruguay, and Venezuela) with data available at the GBD 2019. Using GHDx (11), the age-adjusted mortality per 100 000 individuals and the age-adjusted Disability adjusted Life years (DALYs) per 100 000 individuals for TBL cancer between 1990 and 2019 were obtained. Age-adjusted mortality and DALYs rates are provided together with 95% uncertainty intervals (UI). The 95% uncertainty intervals are determined by the 25th and 75th value of the 1000 values after ordering them from smallest to largest. (15) Absolute numbers and rates (per 100 000 individuals) for 5-year age groups were also obtained. Sex-specific disparities were assessed by obtaining data for females and males. Furthermore, besides analyzing the burden of TBL cancer due to tobacco, we evaluated the contribution of smoking and secondhand smoke to TBL mortality and DALYs.

To evaluate the trends for TBL mortality and DALYs, we calculated the relative percentage change over the study period as:


$$\left[\frac{\text{ age adjusted mortality}\text{ (1990-1994)-}\text{age adjusted mortality}\text{ (2015-2019) }}{\;\text{age adjusted mortality}\text{ (1990-1994) }}\right]*100$$

$$\left[\frac{\;\text{age adjusted DALYs}\text{ (1990-1994)-}\text{age adjusted DALYs}\text{ (2015-2019) }}{\;\text{age adjusted DALYS}\text{ (1990-1994) }}\right]*100$$

